# Personal

**Published:** 1906-02

**Authors:** 


### PERSONAL.

Dr. Roswell Park, of Buffalo, who was very ill for several weeks during January, is convalescent and is resting for a short time at a nearby resort. He expects to resume his professional work at an early day. It is greatly to be regretted that his illness prevented him from attending the Centennial Meeting of the Medical Society of the State of New York and delivering in person his oration on surgery, an abstract of which is published elsewhere.

Dr. James E. King, of Buffalo, announces that he will occupy office until May 1, 1906, with Dr. Nelson G. Russell at 475 Franklin Street. Hours: 11-12 A. M., and by appointment. Telephones: Bell, Tupper 230; Frontier, 930.

Dr. Lucien Howe, of Buffalo, in a communication to the house of delegates, dated January, 29, 1906, offered to create a medical essay prize fund of $1,5000, for the Medical Society of the State of New York, to administer as trustee. The offer was accepted with gratitude, and it was recommended that the grant be known as the Lucien Howe prize fund.

Dr. Francis E. Fronczak, of Buffalo,, delivered an illustrated address before the Buffalo Society of Natural Sciences Friday evening, February 16, 1906, the subject of which was "Poland and her People." Dr. Franczak entertained his large audience in a most interesting manner.

Professor Karl Harko von Noorden, of Frankfort, Germany, lately on a visit to this country, has been appointed by the Minister of Education of Austria as the head of the Vienna Clinic, the place made vacant last summer by the death of Professor Nothnagel.

Dr. H. M. Gaylord announces that he has removed his offices to 695 Delaware Ave., where he will share the offices of Dr. C. Sumner Jones. Hours 3 to 4, and by appointment. Practise limited to surgery.

Dr. Carlos F. MacDonald, of New York, has removed his office from 29 E. 44th Street, to "The Gallatin," 70 W. 46th St. Hours: 10 to 12, Sundays excepted. Telephones: 5514, 3080, and 3081, Bryant.

Dr. Charles W. Pillsbury, of Chicago, is sojourning a few weeks in Buffalo inthe interest of Messrs. Armour & Company. He is accompanied by Mrs. Pillsbury.

